# Spirituality in Coping with Pain in Cancer Patients: A Cross-Sectional Study

**DOI:** 10.3390/healthcare9121671

**Published:** 2021-12-02

**Authors:** Sharon Shyrley Weyll Oliveira, Rayzza Santos Vasconcelos, Verônica Rabelo Santana Amaral, Hélder Fernando Pedrosa e Sousa, Maria Alzira Pimenta Dinis, Diogo Guedes Vidal, Katia Nunes Sá

**Affiliations:** 1 Health Sciences Department, Universidade Estadual de Santa Cruz, Ilhéus 45662-900, Brazil; 2 Medicine Course, Faculdade Santo Agostinho—AFYA, Itabuna 45662-900, Brazil; rayzzauesc@gmail.com (R.S.V.); vekarabelo@gmail.com (V.R.S.A.); 3 Post Graduation Department, Bahiana School of Medicine and Public Health, Salvador 40290-000, Brazil; katia.sa@bahiana.edu.br; 4 Department of Mathematics (DM.UTAD), University of Trás-os-Montes and Alto Douro (UTAD), 5001-801 Vila Real, Portugal; hfps@utad.pt; 5 UFP Energy, Environment and Health Research Unit (FP-ENAS), University Fernando Pessoa (UFP), 4249-004 Porto, Portugal; madinis@ufp.edu.pt (M.A.P.D.); diogovidal@ufp.edu.pt (D.G.V.)

**Keywords:** healthcare, spirituality, coping strategies, pain, cancer

## Abstract

Spirituality has been identified as an adaptive coping strategy and a predictor of better quality of life in cancer patients. Despite the relevance of spirituality in the health–disease process, it is noted that the assessment of the impact of spirituality in coping with pain is still incipient. The objective of this study is to assess the impact of spirituality in coping with pain in cancer patients. This quantitative cross-sectional study was carried out in a medium-sized hospital and a cancer patient support institution located in northeastern Brazil. A questionnaire with sociodemographic and clinical variables was used and the following instruments were applied: Visual Analogue Scale (VAS); Short-Form McGill Pain Questionnaire (SF-MPQ); Neuropathic Pain 4 Questions (DN4); Spiritual Wellbeing Scale (SWBS); WHOQOL Spirituality, Religiousness and Personal Beliefs (WHOQOL-SRPB). Most people with no pain had higher scores on the SWBS. Neuropathic pain was identified in 23 patients and was associated with the highest level of spirituality used as a way of coping with pain. As faith increases, pain decreases in intensity by 0.394 points. On the other hand, as inner peace increases, pain increases by 1.485 points. It is concluded that faith is a strategy for coping with pain, in particular neuropathic pain, minimizing its intensity. On the other hand, greater levels of inner peace allow to increase the awareness of the painful sensation. It is expected that these findings may be useful to integrate spirituality care in healthcare facilities as a resource for positive coping for people in the process of becoming ill, contributing to the therapeutic path and favouring a new meaning to the experience of the disease.

## 1. Introduction

Cancer is characterized by inefficiency in the control of cell division, with a consequent disordered growth of cells which tend to invade healthy tissues and organs of the human body [1]. The patient’s clinical condition and cancer progression are associated with pain, which is a common symptom in patients with malignant neoplasms [2].

Pain is defined by the International Association for the Study of Pain as “an unpleasant sensory and emotional experience associated with, or resembling that associated with, actual or potential tissue damage” [3]. In cancer patients, pain is frequent and usually presents with moderate to severe intensity, requiring the use of morphine for its control, in many cases [4].

Pain assessment is necessary to verify the effectiveness of interventions and understand the temporal behavior of oncologic pain [5]. As it is a subjective phenomenon, the patient’s report is the best measure of intensity [6]. However, some scales, questionnaires and quantitative sensory examination complement pain assessment. Among the dimensions assessed, these tools help to understand the intensity, frequency, duration, location, type and impacts of pain. It is noted, however, that psychosocial–spiritual domains are often neglected in pain assessment, despite the direct or indirect influence on the painful phenomenon [7,8]. Cancer-associated pain is even more complex, as it involves the phenomenon of finitude of life, requiring a multidimensional approach for relief and treatment [9].

Spirituality has been pointed out as an adaptive coping strategy [10] and as a predictor of better quality of life for cancer patients [11]. Spirituality and religiosity are different concepts, yet apparently similar. In fact, they are interdependent and communicating [12], but the religiosity dimension is institutional, based on beliefs and rituals, and is directly related to a certain degree of participation or adherence to religious practices [13]. On the other hand, the spiritual dimension is a broader psychosociological construct [14], with a more individual and subjective character, unfinished and shaped by life experiences.

Despite the relevance of spirituality in the health–disease process of cancer patients, it is noted that the assessment of the impact of spirituality in coping with pain in these patients is still embryonic [15]. Therefore, this study aims to overcome this gap and contribute to the construction of this knowledge. Accordingly, the aim of the study was to assess the impact of spirituality on coping with pain in cancer patients.

## 2. Materials and Methods

This is a quantitative, cross-sectional study, carried out in a medium-sized hospital and a cancer patient support institution located in northeastern Brazil. This hospital is a regional reference for oncological treatment of a nonprofit institution which aims to support adult cancer patients undergoing cancer treatment, assisted by the Unified Health System in a situation of social vulnerability.

The population consisted of people admitted to the hospital with a diagnosis of cancer. In the support institution, the population comprised people undergoing cancer treatment, staying there during the collection period. The inclusion criteria were people aged 18 years or over diagnosed with cancer and who agreed to participate in the study by signing the Informed Consent Form. Exclusion criteria were absence of cancer pain, diagnosis of mental illness, i.e., with dementia or dementia-like symptoms, in the denial phase of the disease, and with weakness or impossibility of communication.

Data collection was carried out by two duly trained and qualified nurses from August 2019 to March 2020. Data collection took place in the hospital bed or on the premises of the support institution. Face-to-face (f2f) interviews were chosen, with the researcher assisting in the application of the instruments’ items without interfering in the interpretation of the answers. Subsequently, clinical data were collected from the medical record.

A questionnaire was used, with information on sociodemographic (age, sex, marital status, education, household and income, religion and health insurance) and clinical variables (type and stage of cancer, diagnosis time, treatment time, current treatment and analgesic use). In addition, the following instruments were used: Visual Analogue Scale (VAS); Short-Form McGill Pain Questionnaire (SF-MPQ); Neuropathic Pain 4 Questions (DN4); Spiritual Wellbeing Scale (SWBS); WHOQOL Spirituality, Religiousness and Personal Beliefs (WHOQOL-SRPB).

The mean pain intensity was assessed using the VAS, obtained from the patient’s report, who assigned a score from 0 to 10 points for the intensity of their pain, with 0 being the absence of pain and 10 being the most intense pain imaginable [16].

The SF-MPQ is an instrument for assessing pain quality, translated and validated by several countries, including Brazil. The Brazilian version of the SF-MPQ, consisting of three dimensions, i.e., sensitive/sensory, affective and evaluative, has 15 descriptors, 8 sensory, 5 affective and 2 evaluative, classified in binary mode (absent or present) [17].

The DN4 instrument helps to differentiate nociceptive pain from neuropathic pain. It was validated for the Portuguese language [18] and consists of 10 items, of which 7 questions address pain symptoms and characteristics obtained through self-report, and 3 refer to physical examination [19]. For each positive answer, a point is given and for each negative item, the score is zero. The total score is calculated as the sum of the 10 items whose cutoff point for the diagnosis of neuropathic pain is the total score of four positive answers (4/10), or without physical examination with three positive answers (3/7) [20]. In the present study, it was decided not to apply the physical examination, considering the cutoff point of 3/7.

The SWBS is a short and easy-to-apply instrument, adapted and validated for the Brazilian context, which can be answered in around five minutes [21]. It is a reference instrument for measuring spirituality. It contains 20 items, and their sum results in the general score of the SWBS. Furthermore, it is divided into two subscales: Religious Wellbeing (RWB) and Existential Wellbeing (EWB) [10].

The WHOQOL-SRPB assesses how spirituality, religion and personal beliefs are related to health-related quality of life [22]. This instrument was validated for Brazil, comprising 32 questions and eight dimensions: connection with being or spiritual strength, meaning in life, admiration, totality and integration, spiritual strength, inner peace, hope and optimism and faith [23]. Each question must be answered on a Likert-type scale, ranging from one to five. The result is expressed through the score of each dimension, so to calculate the scores of the dimensions, the four questions belonging to each dimension must be added and then divided by four [24].

All information collected was tabulated in a Microsoft Excel data sheet and analyzed using the Statistical Package of Social Sciences version 27.0 (IBM, Armonk, NY, USA). Absolute and relative frequencies were calculated for categorical variables and means and standard deviations for quantitative variables. Fisher’s chi-squared test was applied to verify the association between SWBS and SF-MPQ and DN4. Analysis of variance (ANOVA) was applied to assess the existence of differences between the degrees of DN4 and SWBS. Finally, a linear regression analysis was performed to identify the predictor variables of pain (Brief Pain Inventory—BPI 5), as a dependent variable, and WHOQOL-SRPB, as independent variables. The weighting of each independent variable reveals its contribution to the global prediction and helps to understand each variable in predicting the dependent variable [25].

This study was approved by the Ethics and Research Committee of the State University of Santa Cruz, under reference 3.022.500, through CAAE 01564218.2.0000.5526.

## 3. Results

The survey identified 163 potential patients, of which 111 were ineligible, 3 refused to participate and 49 were included in the final sample. According to Table 1, there was a predominance of females (28; 57.1%), mean age 52.3 years (±12.0), marital status married/stable union (23; 46.9%) and people of Catholic religion (21; 42.9%) and Evangelical (21; 42.9%). As for clinical aspects, the most common types of cancer were breast (11; 22.4%) and colorectal (10; 20.4%) and the stage of the disease was not defined in most of the population (25; 51.0%). Most of the sample had known about the diagnosis for ≤12 months (26; 53.1%), was undergoing treatment (30, 62.5%) and radiotherapy treatment (20; 40.8 %). 

Mean pain intensity measured by VAS was 7.9 ± 2.06. Table 2 allows us to verify the association between the SF-MPQ variables and SWBS. The EWB subscale showed a significant association with burning sensation (*p* = 0.019) and suffocating (*p* = 0.040), and most people with no pain had a higher score on the EWB subscale.

Neuropathic pain was identified in 23 patients and was associated with the level of spirituality, which is used as a way of coping (Table 3). Considering these results, it can be stated that there is an association between variables related to neuropathic pain and spirituality, so that when patients experience this pain with description of characteristics such as painful cold, electric shock, itching, they present higher EWB and SWBS total levels, that is, spirituality is used as a way of coping with pain.

In addition, it was observed that patients with a higher level of spirituality had less intense neuropathic pain (Table 4). In both EWB and SWBS, moderate and high levels of wellbeing have lower levels of pain (*p* < 0.05).

Data presented in Table 5 reveal two variables that significantly predict pain: the faith dimension (B = 0.394; *p* < 0.05) and inner peace (B = 1.485; *p* < 0.05). Thus, as faith increases, pain decreases by 0.394 points. On the other hand, as inner peace increases, pain increases by 1.485 points.

## 4. Discussion

In the present study, most people with no pain had higher scores on the EWB subscale, indicating that the mobilization of faith in coping with adverse health situations, such as cancer, can result in a significant reduction in pain intensity. Previous studies confirm that the spiritual dimension and faith can help control pain and suffering, comforting patients. In more advanced stages of the disease, it helps the process of accepting the possibility of death as a natural fact [26,27]. Furthermore, spiritual intervention during treatment helps in the process of pain control and coping with adverse effects of treatment [28].

With regard to the association of pain characteristics with spirituality, most people with no burning and suffocating attributes had greater existential wellbeing. These attributes refer to sensitive aspects of pain and are associated with actual or potential tissue damage [29]. Pain is a subjective vital sign, whose intensity is not only related to the painful stimulus that occurs in the human body, but also to the individual’s cognitive and emotional state [30]. The burning descriptor is generally sensorimotor in nature, while the term suffocating is more associated with the cognitive impact of pain. This finding reveals that existential wellbeing can positively interfere in the biopsychological processing of pain.

Concerning neuropathic pain, it is known that it is a type of chronic pain, arising from lesion or dysfunction of the nervous system, characterized by intense pain that is difficult to be treated [31]. This type of pain tends to decrease the quality of life and affect the performance of activities of daily living. Considering this, it has become increasingly evident that spiritual practices play a relevant role for patients suffering from chronic pain [32], especially when it involves the nervous system.

Neuropathic pain was associated with the level of spirituality, used as a way of coping. Patients who experience this pain with descriptions of characteristics such as painful cold, electric shock and itching, had higher levels of EWB and total SWBS, i.e., spirituality is used as a way of coping with pain. This finding converges with that found in the study by Laluce et al. [33], in which patients with chronic neuropathic pain use spiritual and religious practices as coping strategies, in addition to social support and focusing on the problem.

Participants with a higher level of spirituality had less intense neuropathic pain. According to Ganasegeran et al. [34], the level of pain tolerance seems to be higher in those who sought spiritual resources. The same authors also noted that patients with neuropathic pain had significantly higher dimensions of spirituality than patients with other types of pain, i.e., muscle, inflammatory and mechanical.

As expected, both in the EWB and in the SWBS, those with moderate and high levels of wellbeing have lower levels of pain. This finding confirms the importance of spirituality in coping with cancer patients’ pain, even though there are discrepant results, as seen in the study by Lovell et al. [35], in which persistent pain was associated with low levels of spiritual wellbeing. It is possible that spirituality is influenced by cultural and religious values. If, on the one hand, existential and spiritual wellbeing can strengthen the will to fight cancer, then on the other hand it can lead to blind faith that simply expects a miracle, without adding cognitive coping tasks.

Regarding the quality of life related to spirituality, the dimension faith and inner peace were observed to be predictors of pain in the present study. As faith increases, pain decreases by 0.394 points. On the other hand, as inner peace increases, pain increases by 1.485 points. Therefore, it is believed that physical discomfort and high intensity of pain can reduce inner peace as a protective reaction. The high intensity of pain in cancer cases may also be due to emotional, spiritual and even economic problems, interfering with the quality of life [36]. It is noteworthy that the results presented in this study are in agreement with those found in the meta-analysis by Xing et al. [37], who found that spiritual interventions were able to improve spiritual wellbeing and quality of life, in addition to reducing depression, anxiety and hopelessness in cancer patients.

Although it may seem contradictory, patients who reported having/feeling pain showed inner peace, as well as a search for resignation and a meaning in life. This result is corroborated by previous studies, in which it was observed that the encounter with inner peace was considered as a form of adjustment to the disease situation that the patient is facing. It is expected that in situations of greater gravity, with greater intensity of pain, the feeling of inner peace is greater, as a result of resignation in the face of an adverse situation [38,39,40,41]. It is also possible that, by finding greater inner silence and peace, the person will increase the perception or awareness of the painful sensation, feeling more able to accept and face the problem.

The impact of pain on the quality of life in the spirituality module was confirmed through a moderate degree of correlation. It was found that the 34.5% of the pain is explained by the model. On the other hand, the significance shows that the model can predict pain, being well-adjusted to the data. To this fact the value of the Durbin–Watson test (2.462) is added, confirming that within the range of 1.5 to 2.5 it expresses the absence of autocorrelation.

### Strengths and Limitations

This study has a design that limits the establishment of cause–effect relations, and the fact that no distinction has been performed regarding population cancer stage makes it impossible to associate the pain with the cancer, even though a linear regression has been developed. Additionally, the sample size is reduced and so the results should not be extrapolated to the Brazilian population. The study setting was restricted to a hospital and a reference support institution in the interior region of northeastern Brazil, making it difficult to generalize the findings to large urban centers. Therefore, the replication of the methodological model in other regions and countries is recommended, since cultural and socioenvironmental factors may have influenced the results.

## 5. Conclusions

With the objective to assess the impact of spirituality in coping with pain in cancer patients, this cross-sectional study applied pain and spiritual instruments to a sample in a medium-sized hospital and a cancer patient support institution located in northeastern Brazil. The findings of this study show that high levels of spirituality help in coping with pain in cancer patients. The study’s participants have their inner peace altered and seek resignation and a meaning in life. It is concluded that faith is a strategy for coping with pain, in particular neuropathic pain, minimizing its intensity. The higher levels of inner peace allow the patients to increase their awareness of the painful sensation. Since integrating spiritual care in cancer patients’ treatment stills challenging, it is expected that this study’s findings may provide a contribution and reinforce the fact that this dimension should be assumed as a resource for positive coping for people in the process of becoming ill, contributing to the therapeutic path and favouring a new meaning to the experience of the disease. Future studies should consider the longitudinal influence of socioenvironmental factors.

## Figures and Tables

**Table 1:** Sociodemographic and clinical characterization of cancer patients with pain.

Variable	*n*	%
**Sex**		
Male	21	42.9
Female	28	57.1
**Marital status**		
Married/stable union	23	46.9
Single	15	30.6
Divorced/separated	8	16.3
Widower/widow	3	6.1
**Religion**		
Catholic	21	42.9
Evangelical	21	42.9
Other	3	6.1
None	4	8.2
**Type of cancer**		
Mama	11	22.4
Colorectal	10	20.4
Cervix	8	16.3
Prostate	4	8.3
Stomach and esophagus	8	16.3
Other	8	16.3
**Cancer stage**		
I	1	2.0
II	1	2.0
III	3	6.1
IV	19	38.8
No staging	25	51.0
**How long have you known about your illness?**		
≤12 months	26	53.1
13–24 months	10	20.4
>25 months	13	26.5
**How long ago did you start the treatment?**		
≤12 months	30	62.5
13–24 months	5	10.4
>25 months	14	27.1
**Type of treatment**		
Chemotherapy	12	24.5
Radiotherapy	20	40.8
Surgery	4	8.2
None	13	26.5

Note: Cancer staging was based on the Tumor-Node-Metastasis (TNM) System for Classification of Malignant Tumors. When categories T, N and M are grouped in pre-established combinations, they are distributed in stages that generally vary from I to IV. Such staging was obtained from the medical record.

**Table 2:** Association between pain characteristics according to Short-Form McGill Pain Questionnaire (SF-MPQ) and the Spiritual Wellbeing Scale (SWBS) of cancer patients.

Short-Form McGill Pain Questionnaire		Spiritual Wellbeing Scale								
		RWB			EWB			SWBS		
SF-MPQ		Low	Mod	High	Low	Mod	High	Low	Mod	High
Throbbing	A	1 (100)	1 (16.7)	12 (29.3)	1 (50.0)	7 (20.0)	6 (54.5)	1 (100.0)	2 (12.5)	11 (35.5)
	P	0 (0)	5 (83.3)	29 (70.7)	1 (50.0)	28 (80.0)	5 (45.5)	0 (0)	14 (87.5)	20 (64.5)
	*p*	0.302			0.072			0.075		
Twinge	A	1 (100)	1 (16.7)	9 (22.0)	1 (50.0)	6 (17.1)	4 (36.4)	1 (100)	3 (18.8)	7 (22.6)
	P	0 (0)	5 (83.3)	32 (78.0)	1 (50.0)	29 (82.9)	7 (63.6)	0 (0)	13 (81.3)	24 (77.4)
	*p*	0.251			0.270			0.172		
Shock	A	1 (100)	4 (100)	19 (54.3)	2 (100)	14 (50.0)	8 (80.0)	1 (100)	6 (54.5)	17 (60.7)
	P	0 (0)	0 (0)	16 (45.7)	0 (0.0)	14 (50.0)	2 (20.0)	0 (0)	5 (45.5)	11 (39.3)
	*p*	0.186			0.125			0.667		
Dead/needled	A	1 (100)	4 (66.7)	14 (34.1)	1 (50.0)	12 (34.3)	6 (54.5)	1 (100)	8 (50.0)	10 (32.3)
	P	0 (0)	2 (33.3)	27 (65.9)	1 (50.0)	23 (65.7)	5 (45.5)	0 (0)	8 (50.0)	21 (67.7)
	*p*	0.104			0.465			0.229		
Hook	A	1 (100)	3 (50.0)	13 (31.7)	1 (50.0)	10 (28.6)	6 (54.5)	1 (100)	7 (43.8)	9 (29.0)
	P	0 (0)	3 (50.0)	28 (68.3)	1 (50.0)	25 (71.4)	5 (45.5)	0 (0)	9 (56.3)	22 (71.0)
	*p*	0.269			0.264			0.239		
Burning	A	1 (100)	2 (33.3)	9 (22.0)	1 (50.0)	5 (14.3)	6 (54.5)	1 (100)	2 (12.5)	9 (29.0)
	P	0 (0.0)	4 (66.7)	32 (78,0)	1 (50.0)	30 (85.7)	5 (45.5)	0 (0)	14 (87.5)	22 (71.0)
	*p*	0.180			0.019			0.100		
Spread	A	1 (100)	2 (33.3)	20 (48.8)	1 (50.0)	15 (42.9)	7 (63.6)	1 (100)	7 (43.8)	15 (48.4)
	P	0 (0.0)	4 (66.7)	21 (51.2)	1 (50.0)	20 (57.1)	4 (36.4)	0 (0)	9 (56.3)	16 (51.6)
	*p*	0.447			0.484			0.549		
Painful	A	0 (0.0)	1 (16.7)	1 (2.4)	0 (0.0)	1 (2.9)	1 (9.1)	0 (0.0)	1 (6.3)	1 (3.2)
	P	1 (100)	5 (83.3)	40 (97.6)	2 (100)	34 (97.1)	10 (90.9)	1 (100)	15 (93.8)	30 (96.8)
	*p*	0.260			0.636			0.867		
Tiresome	A	0 (0.0)	2 (33.3)	5 (12.2)	0 (0.0)	3 (8.6)	4 (36.4)	0 (0.0)	3 (18.8)	4 (12.9)
	P	1 (100)	4 (66.7)	36 (87.8)	2 (100)	32 (91.4)	7 (63.6)	1 (100)	13 (81.3)	27 (87.1)
	*p*	0.358			0.062			0.793		
Sick	A	0 (0.0)	2 (33.3)	8 (19.5)	0 (0.0)	6 (17.1)	4 (36.4)	0 (0.0)	6 (37.5)	4 (12.9)
	P	1 (100)	4 (66.7)	33 (80.5)	2 (100)	29 (82.9)	7 (63.6)	1 (100)	10 (62.5)	27 (87.1)
	*p*	0.646			0.298			0.126		
Suffocating	A	0 (0.0)	2 (33.3)	14 (34.1)	0 (0.0)	9 (25.7)	7 (63.6)	0 (0.0)	7 (43.8)	9 (29.0)
	P	1 (100)	4 (66.7)	27 (65.9)	2 (100)	26 (74.3)	4 (36.4)	1 (100)	9 (56.3)	22 (71.0)
	*p*	0.774			0.040			0.463		
Terrifying	A	0 (0.0)	2 (33.3)	9 (22.0)	0 (0.0)	6 (17.1)	5 (45.5)	0 (0.0)	5 (31.3)	6 (19.4)
	P	1 (100)	4 (66.7)	32 (78.0)	2 (100)	29 (82.9)	6 (54.5)	1 (100)	11 (68.8)	25 (80.6)
	*p*	0.709			0.110			0.563		
Bored	A	0 (0.0)	2 (33.3)	8 (19.5)	0 (0.0)	7 (20.0)	3 (27.3)	0 (0.0)	6 (37.5)	4 (12.9)
	P	1 (100)	4 (66.7)	33 (80.5)	2 (100)	28 (80.0)	8 (72.7)	1 (100)	10 (62.5)	27 (87.1)
	*p*	0.646			0.664			0.126		
Troublesome	A	0 (0.0)	1 (16.7)	1 (2.4)	0 (0.0)	1 (2.9)	1 (9.1)	0 (0.0)	1 (6.3)	1 (3.2)
	P	1 (100)	5 (83.3)	40 (97.6)	2 (100)	34 (97.1)	10 (90.9)	1 (100)	15 (93.8)	30 (96.8)
	*p*	0.260			0.636			0.867		
Unbearable	A	0 (0.0)	2 (33.3)	5 (12.2)	0 (0.0)	3 (8.6)	4 (36.4)	0 (0.0)	3 (18.8)	4 (12.9)
	P	1 (100)	4 (66.7)	36 (87.8)	2 (100)	32 (91.4)	7 (63.6)	1 (100)	13 (81.3)	27 (87.1)
	*p*	0.358			0.062			0.793		

Note: RWB = Religious Wellbeing; EWB = Existential Wellbeing; SWBS = Spiritual Wellbeing Scale; A = Absent; P = Present; Mod = Moderate; *p* = Chi square *p*-value.

**Table 3:** Association of neuropathic pain with spirituality levels according to the Spiritual Wellbeing Scale (SWBS).

		Spiritual Wellbeing Scale								
Neuropathic Pain		RWB			EWB			SWBS Total		
		Low	Mod	High	Low	Mod	High	Low	Mod	High
Burning	N	0 (0.0)	4 (66.7)	32 (76.2)	1 (50.0)	28 (77.8)	7 (63.6)	0 (0.0)	11 (68.8)	25 (78.1)
	Y	1 (100)	2 (33.3)	10 (23.8)	1 (50.0)	8 (22.2)	4 (36.4)	1 (100)	5 (31.3)	7 (21.9)
	*p*	0.215			0.483			0.191		
Painful cold	N	1 (100)	2 (33.3)	12 (28.6)	1 (50.0)	14 (38.9)	0 (0.0)	1 (100)	8 (50.0)	6 (18.8)
	Y	0 (0.0)	4 (66.7)	30 (71.4)	1 (50.0)	22 (61.1)	11 (100)	0 (0.0)	8 (50.0)	26 (81.3)
	*p*	0.306			0.041			0.027		
Electric shock	N	1 (100)	1 (16.7)	11 (26.2)	2 (100)	10 (27.8)	1 (9.1)	1 (100)	6 (37.5)	6 (18.8)
	Y	0 (0.0)	5 (83.3)	31 (73.8)	0 (0.0)	26 (72.2)	10 (90.9)	0 (0.0)	10 (62.5)	26 (81.3)
	*p*	0.215			0.026			0.093		
Tingling	N	1 (100)	3 (50.0)	19 (45.2)	2 (100)	20 (55.6)	1 (9.1)	1 (100)	10 (62.5)	12 (37.5)
	Y	0 (0.0)	3 (50.0)	23 (54.8)	0 (0.0)	16 (44.4)	10 (90.9)	0 (0.0)	6 (37.5)	20 (62.5)
	*p*	0.548			0.008			0.147		
Needled and needled	N	0 (0.0)	4 (66.7)	28 (66.7)	1 (50.0)	24 (66.7)	7 (63.6)	0 (0.0)	11 (68.8)	21 (65.6)
	Y	1 (100)	2 (33.3)	14 (33.3)	1 (50.0)	12 (33.3)	4 (36.4)	1 (100)	5 (31.3)	11 (34.4)
	*p*	0.383			0.883			0.374		
Falling asleep	N	1 (100)	2 (33.3)	21 (50.0)	1 (50.0)	18 (50.0)	5 (45.5)	1 (100)	10 (62.5)	13 (40.6)
	Y	0 (0.0)	4 (66.7)	21 (50.0)	1 (50.0)	18 (50.0)	6 (54.5)	0 (0.0)	6(37.5)	19 (59.4)
	*p*	0.439			0.965			0.212		
Itch	N	1 (100)	3 (50.0)	13 (31.0)	2 (100)	14 (38.9)	1 (9.1)	1 (100)	8 (50.0)	8 (25.0)
	Y	0 (0.0)	3 (50.0)	29 (69.0)	0 (0.0)	22 (61.1)	10 (90.9)	0 (0.0)	8 (50.0)	24 (75.0)
	*p*	0.251			0.027			0.088		
Categorized neuropathic pain	N	0 (0.0)	3 (50.0)	23 (54.8)	0 (0.0)	16 (44.4)	10 (90.9)	0 (0.0)	5 (31.3)	21 (65.6)
	Y	1 (100)	3 (50.0)	19 (45.2)	2 (100)	20 (55.6)	1 (9.1)	1 (100)	11 (68.8)	11 (34.4)
	*p*	0.548			0.008			0.045		

Note: RWB = Religious Wellbeing; EWB = Existential Wellbeing; SWBS = Spiritual Wellbeing Scale; N = No; Y = Yes; Mod = Moderate; *p* = Chi square *p*-value.

**Table 4:** Association between neuropathic pain intensity and spirituality levels according to the Spiritual Wellbeing Scale (SWBS).

Spiritual Wellbeing Scale	Total Neuropathic Pain					F; p
	*N*	Mean	Standard Deviation	Minimum	Maximum	
**RWB**						
Low	1	6.00	n.a.	6	6	1.151; 0.325
Moderate	6	3.67	1.75	1	6	
High	42	3.38	1.74	0	7	
Total	49	3.47	1.75	0	7	
**EWB**						
Low	2	6.00	0.000	6	6	8.657; 0.001
Moderate	36	3.78	1.62	1	7	
High	11	2.00	1.18	0	4	
Total	49	3.47	1.74	0	7	
**SWBS**						
Low	1	6.00	n.a.	6	6	5.588; 0.007
Moderate	16	4.38	1.82	1	7	
High	32	2.94	1.48	0	6	
Total	49	3.47	1.74	0	7	

Note: RWB = Religious Wellbeing; EWB = Existential Wellbeing; SWBS = Spiritual Wellbeing Scale; n.a. Not applicable.

**Table 5:** Linear regression of the predictor variable pain and the impact of quality-of-life module spirituality.

Model	B	S. Error	Beta	*t*	*p*
(Constant)	2.722	3.800		0.716	0.479
Faith	−0.394	0.187	−0.668	−2.103	0.043
Connection with a spiritual being or force	0.957	1.077	0.195	0.888	0.381
Meaning in life	−0.910	0.744	−0.262	−1.223	0.230
Admiration	−0.004	0.563	−0.001	−0.007	0.995
Full integration	−0.441	0.663	−0.135	−0.665	0.510
Spiritual strength	0.914	0.790	0.320	1.156	0.256
Inner peace	1.485	0.531	0.492	2.800	0.008
Hope and optimism	0.828	0.787	0.329	1.053	0.300

Note: *R* = 0.587; *R*^2^ =0.345; *R*^2^_a_ =0.191; *F* = 2.237; *p* < 0.05; Durbin-Watson = 2.462.

## Data Availability

Data is available upon request to the corresponding author.
